# Potential Roles of Adropin in Central Nervous System: Review of Current Literature

**DOI:** 10.3389/fmolb.2016.00025

**Published:** 2016-06-27

**Authors:** Shima Shahjouei, Saeed Ansari, Tayebeh Pourmotabbed, Ramin Zand

**Affiliations:** ^1^Department of Neurosurgery, Tehran University of Medical SciencesTehran, Iran; ^2^Department of Neurology, University of Tennessee Health Science CenterMemphis, TN, USA; ^3^Department of Microbiology, Immunology, and Biochemistry, University of Tennessee Health Science CenterMemphis, TN, USA; ^4^Biocomplexity Institute, Virginia Polytechnic Institute and State UniversityBlacksburg, VA, USA

**Keywords:** adropin, neurodegenerative disease, neuroprotection, biomarker, predictor, therapeutic, cellular signaling pathways

## Abstract

Adropin is a 4.9 kDa peptide that is important for maintenance of metabolic and non-metabolic homeostasis. It regulates glucose and fatty acid metabolism and is involved in endothelial cell function and endothelial nitric oxide (NO) synthase bioactivity as well as physical activity and motor coordination. Adropin is expressed in many tissues and organs including central nervous system (CNS). This peptide plays a crucial role in the development of various CNS disorders such as stroke, schizophrenia, bipolar disorder as well as Alzheimer's, Parkinson's, and Huntington's diseases. In this comprehensive review, the potential roles of adropin in cellular signaling pathways that lead to pathogenesis and/or treatment of CNS disorders will be discussed.

## Introduction

Adropin is a 4.9 kDa peptide encoded by Energy Homeostasis Associated gene (*Enho*) located on chromosome 9 (Kumar et al., 2008; Aydin, 2014). A variety of organs including central nervous system (neurons, neuroglial cells, pia mater, vascular area, Purkinje cells, and granular layer), heart, kidney, liver, pancreas, and human umbilical vein synthesize adropin (Lovren et al., 2010; Aydin et al., 2013, 2014).

**Figure 1:**
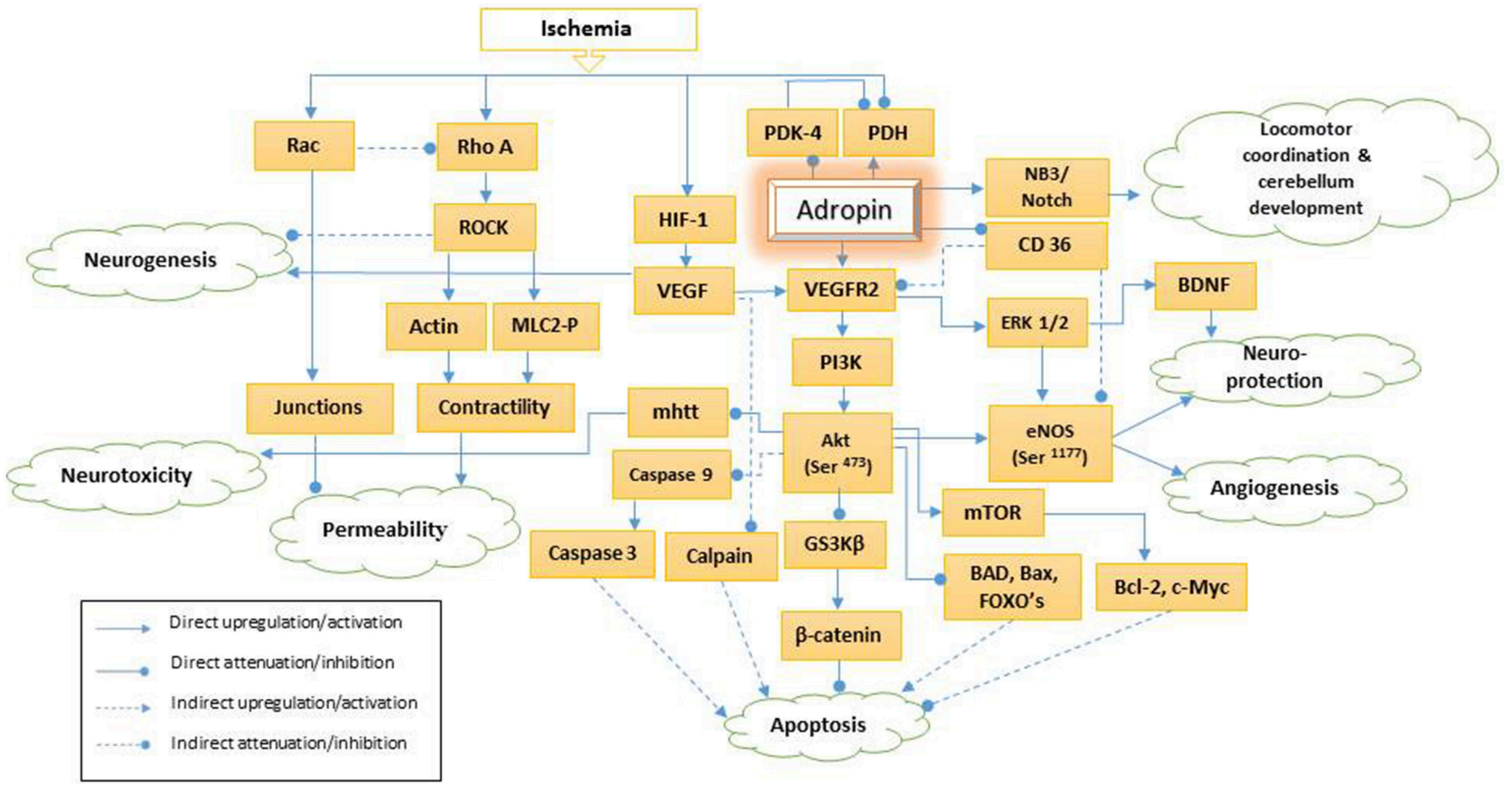
**Schematic presentation of adropin signaling pathways**. PDH, pyruvate dehydrogenase; PDK-4, pyruvate dehydrogenase kinase-4; HIF-1, hypoxia-inducible factor-1a; NB3, neural recognition molecule 3; VEGF, vascular endothelial growth factor; mhtt, mutant huntingtin; VEGFR2, vascular endothelial growth factor receptor 2; PI3K, phosphatidylinositol-3 kinase; Akt (Ser-473), phosphorylation of serine 473 of serine, threonine kinase; GS3Kβ, glycogen synthase kinase 3β; CD36, Cluster of Differentiation36; ERK 1/2, extracellular signal-regulated kinases 1/2; Mtor, mammalian target of rapamycin; BAD, Bcl-2 associated death protein; eNOS (Ser-1177), phosphorylation of endothelial nitric synthase on serine 1177; BNDF, brain-derived neurotrophic factor; FOXO, Forkhead box O; Bcl-2: B-cell lymphoma 2; Bax, Bcl-2 associated X protein; ROCK, Rho-associated protein kinase; MLC2-P, Phosphorylated myosin light chain 2.

Constantly new functions for adropin are identified. Adropin's function as a regulator of glucose and lipid homeostasis and insulin sensitivity was initially described in 2008 by Kumar et al. (2008) and later by Aydin (2014). Lovren et al. (2010) demonstrated the endothelial protective potentials of adropin in 2010. Adropin activates vascular endothelial growth factor receptor 2 (VEGFR2) and its two downstream signaling pathways—phosphatidylinositol-3 kinase/ serine, threonine kinase (PI3K/Akt) and extracellular signal-regulated kinases 1/2 (ERK 1/2) (Figure 1). Therefore, adropin modulates expression of endothelial nitric oxide synthase (eNOS) (Lovren et al., 2010). Also, adropin increases the endothelial cells proliferation, migration and potential to form capillary-like structures (Lovren et al., 2010). Recently, it is found that adropin reduces the endothelial permeability (Lovren et al., 2010; Yang et al., 2016).

Adropin enhances mitochondrial function and activates pyruvate dehydrogenase (PDH)—a rate-limiting enzyme in glucose oxidation. Further, adropin suppresses two key enzymes in fatty acid utilization: carnitine palmitoyltransferase-1B (CPT-1B) and Cluster of Differentiation 36 (CD36) (Gao et al., 2015); thus, it plays a role in fatty acid oxidation.

Adropin may act as a potential protective regulator of atherogenesis and cardiovascular diseases (Wu et al., 2014; Zhao et al., 2015b; Li et al., 2016). Serum adropin level is inversely associated with severity of coronary atherosclerosis and serum level of homocysteine—a potential risk factor for atherosclerosis and cardiovascular diseases (Zhao et al., 2015a). The serum adropin level is diminished in patients with cardiac syndrome X and stable coronary artery disease (Celik et al., 2013; Zhao et al., 2015b). At the onset of acute myocardial infarction, serum adropin level is usually lower than controls (Yu et al., 2014); however, it raises between 1 and 24 h following myocardial infarction (Aydin et al., 2014). Plasma adropin level has a positive association with severity of heart failure and negative correlation with left ventricular ejection fraction (Lian et al., 2011). Low level of plasma adropin is predictive of pseudoexfoliation (Oğurel et al., 2016), coronary slow flow phenomenon (Demircelik and Kurtul, 2015), saphenous vein graft occlusion following coronary artery bypass grafting (Demircelik, 2014), as well as pediatric obstructive sleep apnea in the presence of endothelial dysfunction (Gozal et al., 2013). While Gu et al. (2015) described plasma adropin level as an independent indicator of hypertension, other studies failed to show this association (Altincik and Sayin, 2015).

Adropin, as a membrane-anchored protein modulates the Notch1 signaling pathway via neural recognition molecule 3 (NB3) (Figure 1). NB-3 belongs to the contactin family and acts as a membrane-tethered Notch1 ligand that mediates cell surface interaction during nervous system development. An animal study demonstrated that adropin regulates locomotor activity and motor coordination via the NB3/Notch signaling pathway and plays an important role in cerebellum development (Wong et al., 2014). In this review, we discuss various roles of adropin in central nervous system pathogenesis via different intra and extra cellular signaling pathways as well as its therapeutic potentials.

### Adropin and vascular endothelial growth factor receptor 2 (VEGFR2)

VEGFR2—a tyrosine kinase receptor—is especially expressed in endothelial cells and regulates endothelial function and angiogenesis. Adropin strongly upregulates this receptor, activates PI3K/Akt and ERK1/2 pathways, and enhances eNOS thus, modulating NO bioavailability (Lovren et al., 2010; Figure 1). Hypoxic insults enhance hypoxia-inducible factor-1a (HIF-1a), and VEGF gene expression as its downstream signaling pathway (Mu et al., 2003). VEGF is involved in neurogenesis and has a neuroprotection function. This has been discussed under “Adropin and Neurogenesis” section in more details.

### Role of adropin in activation of PI3K/Akt signaling pathway

PI3K induces the phosphorylation of Akt (also known as protein kinase B) under the effect of growth factors such as VEGF, cytokines, insulin, and other cellular stimuli (Figure 1). Activation of Akt requires consequent phosphorylation on Thr-308 and Ser-473. Once Ser-473 is phosphorylated, Akt is fully activated regardless of Thr-308 phosphorylation status (Wang et al., 2009). Adropin can activate Akt by stimulating Ser-473 phosphorylation (Lovren et al., 2010).

Phosphorylated-Akt provokes cell cycle progression, proliferation, differentiation, and survival (Blanco-aparicio et al., 2007; Manning and Cantley, 2007). Moreover, this pathway triggers intracellular ligands such as mammalian target of rapamycin (mTOR)—which plays an important role in angiogenesis, neuronal regeneration, synaptic plasticity, inflammatory responses, and apoptosis (Annovazzi et al., 2009; Chen et al., 2012; Li et al., 2015). Thereby, PI3K/Akt/mTOR pathway may be a target of stroke therapeutic agents (Li et al., 2015).

Neurodegenerative conditions such as Alzheimer's, Parkinson's and Huntington's diseases are associated with defective Akt signaling (Colin et al., 2005; Griffin et al., 2005; Timmons et al., 2009; Giralt et al., 2010). Similarly, damaged Akt/GSK3β (the serine/threonine kinase glycogen synthase kinase 3β) signaling pathway plays a role in the pathophysiology of neuropsychiatric disorders such as schizophrenia and bipolar disorders (Emamian et al., 2004; Jope, 2011). Since, variation in AKT1—one of the three genes encoding Akt—has been associated with schizophrenia and bipolar disorders (Ikeda et al., 2004; Karege et al., 2012), PI3K/Akt activation by adropin might also have a therapeutic potential in disorders such as Parkinson's (Burke, 2007; Timmons et al., 2009) and schizophrenia (Schwab et al., 2005) as discussed below:

#### Ischemic insult

Cerebral ischemic injuries cause neural loss secondary to apoptosis or necrosis—which can be triggered by oxidative stress, metabolic compromise and disruption of calcium homeostasis at the cellular level (Mattson et al., 2001). Altintas et al. demonstrated that infarct size is positively correlated with blood adropin level in animal models of cerebral ischemia (Altintas et al., 2016). Activation of Akt by adropin can prevent neuronal and cellular death, (Chong et al., 2005) and might contribute to neuro-protective effect of ischemic postconditioning (Gao et al., 2008; Wang et al., 2009). PI3K/Akt pathway induces mTOR and also attenuates apoptotic proteins such as GSK3β and forkhead family of transcription factor. Thereby, inactivation of Akt might contribute to neuronal apoptosis and pathogenesis of ischemic stroke (Noshita et al., 2001; Franke et al., 2003; Hanumanthappa et al., 2014; Li et al., 2015).

#### Huntington disease (HD)

Abnormal expansion of a polyglutamine stretch in the N terminus of protein huntingtin is responsible for neuropathology of HD (Humbert et al., 2002). Induction of Akt Ser-473 phosphorylation attenuates mutant huntingtin toxicity and makes the cell more resistance to apoptotic signals by modulating proteins such as GSK3β and FOXO1 (Humbert et al., 2002; Manning and Cantley, 2007). In addition, activated Akt decreases intranuclear inclusions of mutant huntingtin (Humbert et al., 2002). It was demonstrated that maintaining high levels of activated Akt may delay cell death and allow the recovery of neuronal viability after mutant huntingtin silencing (Canals, 2004).

#### Parkinson's disease (PD)

Timmons and colleagues showed that Akt is expressed at high levels in tyrosine hydroxylase dopaminergic neurons. Selective loss of these neurons and diminished phosphorylated Akt at Ser-473 is obvious in the brain of patients with Parkinson's disease (Timmons et al., 2009). The glial cell line-derived neurotrophic factor (GDNF) as the downstream of phosphorylated Akt has neuroprotective effect against dopaminergic neurodegeneration (Ries et al., 2006). Thus, medications like adropin that target the dopaminergic system via Akt activation or those with the potential to increase the phosphorylated Akt have neuroprotective characteristics in PD (Ries et al., 2006; Burke, 2007; Levy et al., 2009; Timmons et al., 2009).

#### Schizophrenia

AKT1 gene single nucleotide polymorphisms (SNPs) and haplotype studies indicated the involvement of Akt in Schizophrenia (Ikeda et al., 2004; Schwab et al., 2005; Thiselton et al., 2008). Expression or activity of AKT1 and phosphorylation of its substrate—GSK3β—is reduced in Schizophrenic patients (Emamian et al., 2004; Kalkman, 2006). As summarized by Beaulieu and colleagues, many of the antipsychotics and psychoactive substances modulate dopamine-dependent behaviors through Akt/GSK3β signaling pathway (Beaulieu et al., 2007). In addition, Schizophrenia is associated with insulin receptor deficit, disruptive insulin dependent Akt signaling and insulin resistance (Zhao et al., 2006). Adropin might be a potent therapeutic agent in Schizophrenia while it enhances Akt phosphorylation (Lovren et al., 2010) and prevents insulin resistance (Ganesh Kumar et al., 2012).

#### Alzheimer's disease (AD)

Activation of PI3K/Akt/Wnt/β-catenin signaling induces neurogenesis and reverse cognitive deficit in AD animal models (Tiwari et al., 2015). In addition, reduced phospho-Akt and increased FOXO3a levels in the nuclei of neurons where proapototic genes were activated can cause adipokine dyshomeostasis, oxidative stress, mitochondrial dysfunction, and eventually neurodegeneration (Nuzzo et al., 2015). These data suggest Akt might be the link between insulin resistance, obesity, and AD.

#### Bipolar disorder

Regulation of Akt/mTOR pathway is critical in synaptic neurotransmission and plasticity, as well as modulating cell proliferation and migration. There is evidence of excitotoxicity, neuroinflammation, and brain atrophy in BD due to apoptosis and disturbed synaptic function. A cadaver study on BD postmortem prefrontal cortex demonstrated an elevation in protein and mRNA levels of the pro-apoptotic factors (Bax, BAD, caspase-9 and caspase-3) and reduction in anti-apoptotic factors (BDNF and Bcl-2) and the synaptic markers (synaptophysin and drebrin) (Kim et al., 2010). The Bax/Bcl-2 ratio appeared to be crucial in deciding the life or death of a cell and was increased in the above study. In another study, blood AKT1and mTOR mRNA expression decreased in BD during depressive episodes comparing to healthy controls, supporting an integrated Akt/mTOR signaling pathway activity in the pathogenesis of BD (Machado-Vieira et al., 2015). In accordance, activation of mTOR by N-methyl-D-aspartate (NMDA) antagonists results in rapid antidepressant effect in animal models (Li et al., 2010).

Study on animals under high-fat diet showed that obesity may desensitize serotonin-dependent Akt/GSK3β signaling and impair cell proliferation in the dentate gyrus of the hippocampus, and cause depression (Papazoglou et al., 2014). Available evidence support the notion that enhancing the inhibitory control of Akt/GSK3β is a key component of the therapeutic actions of drugs used to treat mood disorders (Li and Jope, 2010).

### Adropin and extracellular signal-regulated kinases 1/2 (ERK1/2)

ERK 1/2 is a member of the mitogen-activated protein kinase family. Adropin via VEGFR2 can activate ERK 1/2 and its downstream cascades of substances such as brain-derived neurotrophic factor (BDNF) (Figure 1; Lovren et al., 2010). BNDF promotes neuronal development, differentiation, survival and neurological function improvement following brain injury and ischemia (Zhu et al., 2013; Zhao et al., 2014; Wu et al., 2015). Ischemic postconditioning, both early and delayed, may further reduce reperfusion injury via ERK 1/2 and BDNF activation (Wu et al., 2015). In contrast, post-ischemic inhibition of ERK 1/2 in diabetic rats may mitigate DNA repairing ability, accelerated apoptosis and aggravate neuronal loss (Zhao et al., 2014). In addition, ERK 1/2 activation induces nuclear factor erythroid 2-related factor2 (Nrf2) and protects neurons against beta-amyloid-induced cell death and oxidative stress.

### Adropin and nitric oxide synthase (NOS)

One of the endothelial protective functions of adropin is regulation of nitric oxide (NO) bioavailability (Lovren et al., 2010). NO promotes angiogenesis, reparative vasculogenesis and acts as an anti-atherosclerotic, anti-inflammatory and anti-thrombotic factor.

NO is generated by nitric oxide synthase (NOS) that is upregulated by PI3K/Akt and ERK 1/2 signaling pathways (Figure 1) (Lovren et al., 2010; Peng et al., 2012). NOS polymorphisms and diminished endothelial NOS expression are associated with spontaneous cerebral thrombosis and infarction, progressive cerebral amyloid angiopathy, blood brain barrier breakdown, and cognitive impairment—characteristics of cerebral small vessel disease, stroke and neurodegenerative diseases such as Alzheimer's disease (Hassan, 2004; Jeynes and Provias, 2009; Tan et al., 2015). Additionally, Tan et al. evidenced that this vascular occlusion occurs exclusively at the same hypoperfused areas identified in preclinical Alzheimer's disease (temporoparietal and retrosplenial granular cortexes, and hippocampus; Tan et al., 2015).

Adropin directly upregulates NOS expression in both *in-vivo* and *in-vitro* endothelial cells resulting in proliferation, migration, and capillary-like tube formation and diminished permeability and apoptosis of these cells (Lovren et al., 2010). Moreover, upregulation of NOS increases cerebral blood flow and prevents stress-induced hypotension, inflammation, apoptosis and cerebral ischemia (Lin et al., 2010). Thus, early administration of nitric oxide or its precursor to patients with acute stroke has been shown to affect lesion size, cerebral blood flow, mood, cognition and quality of life (Willmot et al., 2005; Woodhouse et al., 2015).

### Adropin and cluster of differentiation 36 (CD36)

CD36 is a member of the class B scavenger receptor family and is activated by various ligands with diverse cellular responses—such as the production of free radicals, induction of inflammatory responses, and endothelial dysfunction (Cho, 2005, 2012). CD36 has anti-angiogenic nature and downregulates VEGFR2 phosphorylation, (Primo et al., 2005) and through its ligands such as oxLDL (a major factor in the development of atherosclerosis) causes endothelial cell stiffness and atherosclerosis (Shentu et al., 2010). Adropin downregulates CD36 gene expression and cell surface CD36 protein levels which indicate a potential reduction of muscle fatty acid uptake (Gao et al., 2015). Alongside, adropin treatment has been shown to downregulate peroxisome proliferator-activated receptor-gamma coactivator-1α (PGC-1α) that regulates expression of CD36 (Gao et al., 2015).

CD36 is known to be one of the underlying causes of cerebrovascular and neurodegenerative diseases. Accumulation of β-Amyloid (a CD36 ligand) in the vicinity of plaques of Alzheimer's disease, and in the cerebrovascular wall of hemorrhagic stroke had been described (Winkler et al., 2001; Hernandez-Guillamon et al., 2012). Increased CD36 gene expression following blood-brain barrier damage and circulating amyloid β protein following ischemic insult might contribute to the pathogenesis of vascular dementia and bridge the gap between vascular dementia and Alzheimer's disease (Lee et al., 2005; Ueno et al., 2016).

### Adropin and glucose oxidation

Adropin upregulates glucose oxidation via decreasing acetylation of pyruvate dehydrogenase complex (PDHC, a rate-limiting enzyme in glucose oxidation) and down-regulating pyruvate dehydrogenase kinase-4 (PDK-4)- a PDHC inhibitor. PDHC is a mitochondrial matrix enzyme complex that catalyzes oxidative decarboxylation of pyruvate to produce acetyl-CoA, which plays a critical role in cerebral aerobic energy metabolism (Cardell et al., 1989; Martin et al., 2005). Impaired cerebral energy metabolism and PDHC activity are seen in acute brain injury and chronic neurodegenerative conditions such as Alzheimer's disease and Wernicke-Korsakoff syndrome (Martin et al., 2005). PDHC activity is attenuated after brain ischemia (Cardell et al., 1989; Martin et al., 2005). This reperfusion dependent suppression might be due to the depressed activity of pyruvate dehydrogenase phosphatase or oxidative stress (because of hyperoxic resuscitation) (Martin et al., 2005). Inactivation of PDHC can be a possible cause of post-ischemic metabolic depression, prolonged intracellular lactic acidosis, and secondary tissue energy depletion, which contribute to neuronal injury and neurological impairment (Cardell et al., 1989; Martin et al., 2005). In addition, compensating the enzyme activity by administration of acetyl-L-carnitine which is converted to acetyl-Co or dichloroacetate (DCA) improves neurologic outcome (Rosenthal et al., 1992; Martin et al., 2005). Adropin treatment in animal studies increases the ratio of CoA/acetyl-CoA which directly promote PDHC activity and pyruvate oxidation (Gao et al., 2015).

### Adropin and endothelial permeability

The involvement of Adropin in endothelial permeability was originally described by Lovren and coworkers in 2010 (Lovren et al., 2010). Adropin attenuates the hypoxic/low glycemic induced paracellular permeability by inhibiting ROCK/MLC2 signaling pathway (Figure 1; Yang et al., 2016). As described by Wojciak-Stothard and Ridley, the endothelial permeability is determined by intercellular junctions integrity and basal intracellular actinomyosin contractility (Wojciak-Stothard and Ridley, 2002). Rho GTPases such as Rac 1 and Rho A act antagonistically to regulate endothelial permeability (Wojciak-Stothard and Ridley, 2002; Wojciak-Stothard et al., 2006). Rac 1 enhances the cellular junction and adherence, (Wojciak-Stothard et al., 2006) and inhibits Rho under chronic ischemia (Wojciak-Stothard et al., 2005). In contrast, Rho A and its downstream Rho-associated protein kinase (ROCK) enhance the marginal cell isometric tension and actinomysin contractility (Wojciak-Stothard et al., 2006). Hypoxic/hypoglycemic condition induces activation of Rho/ROCK signaling pathway by stimulating K-ras effector pathways independent of HIF-1 (Mizukami et al., 2006; Wojciak-Stothard et al., 2006; Yang et al., 2016) (Figure 1). Activated ROCK promotes direct phosphorylation of myosin light chain 2 (MLC2) at Ser19 site and inhibition of myosin light chain phosphatase (MLCP). Phosphorylated MLC2 enhances actinomyosin contractility, intracellular tension and increases cellular permeability (Yang et al., 2016). In addition, down regulation of Rac 1 induces actin formation via Rho activation and intensifies contractility (Wojciak-Stothard et al., 2006; Weidemann et al., 2013).

### Adropin and neurogenesis

Induction of mesenchymal cells with inhibitors of prolyl hydroxylase—a key enzyme in HIF-1α degradation—promotes mesenchymal cells differentiation to morphologically neuron-like cells (Pacary et al., 2006). HIF-1α production under ischemic conditions induces potentially neurogenic factors—EPO (erythropoietin), p21 and VEGF (Jin et al., 2002; Yu et al., 2002; Pacary et al., 2006). Animal models of ischemic stroke demonstrated functions for VEGF in neuroprotection (better neurological outcomes and smaller infarct volume), neurogenesis (in both early and delayed phases in neuronal precursors) and in angiogenesis (endothelial cell proliferation, migration, survival and vascular permeability) (Jin et al., 2001; Sun et al., 2003; Shimotake et al., 2010). Although neurogenesis and angiogenesis are known to be coupled, the neurotrophic potential of VEGF might be independent of angiogenesis: VEGF induces axonal outgrowth—by acting on growing axons and nerve cell bodies—and suppresses the cell-death pathways mediated by calpain-dependent and caspase-3-dependent mechanisms (Sondell et al., 2000; Jin et al., 2001; Shimotake et al., 2010).

Recent studies demonstrated that inhibition of Rho/ROCK signaling pathway enhances HIF-1 activity and upregulates EPO, VEGF and p21, and consequently potentiates neurogenesis (Pacary et al., 2007, 2008). Adropin might be a novel candidate to promote neurogenesis as it can inhibit the Rho/ ROCK pathway without affecting VEGF level (Yang et al., 2016).

### Adropin and orphan G protein-coupled receptor (GPR19)

Stein et al. discovered GPR19 as a potential adropin receptor (Stein et al., 2016). GPR19 is a transmembrane receptor similar to the neuropeptide Y receptors and the dopamine D2 receptor family (O'Dowd et al., 1996). GPR19 is more likely expressed in cerebellum, caudate, putamen, thalamus, hypothalamus, hippocampus, frontal cortex and olfactory bulb (O'Dowd et al., 1996; Hoffmeister-Ullerich et al., 2004). Transcripts of GPR19 can be detected in neuroectodermal origin tissues in early embryogenesis, and they are gradually restricted to the regions of the developing brain (Hoffmeister-Ullerich et al., 2004). Signal transduction through GPR19 enhances ERK and Akt phosphorylation in cerebral neurons (Hossain et al., 2016). Recently, Stein et al. described the adropin function in water intake inhibition through GPR19 (Stein et al., 2016). However, the distribution of GPR19 and potency of its downstream signaling pathways suggest more critical actions for adropin in neuronal development and protection.

## Conclusion

Studies regarding the effects of adropin in different organs are still in infancy stage, but increasing evidence suggest that this peptide has unique effects on endothelial cell function via upregulating eNOS expression through the VEGFR2-PI3K-Akt, VEGFR2-ERK 1/2 pathways and inhibition of Rho/ROCK pathway. However, our current knowledge mainly comes from animal studies or treatment with the putative secreted domain of adropin. Whether these findings are transferable to clinical studies needs to be determined. Moreover, adropin may be utilized as a promising biomarker for CNS disease risk stratification or diagnosis, and/or a potential therapeutic candidate in CNS injuries. Although adropin seems to be a novel target to limit vascular diseases, in parallel with the documented effects on metabolic modulation, further investigations are needed to elucidate the specific mechanism underlying the association between adropin and CNS diseases.

## Author contributions

Study concept and design: SA, RZ. Acquisition of data: SS, SA, TP, RZ. Drafting and critical revision of manuscript: SS, SA, TP, RZ. Study supervision: TP, RZ.

### Conflict of interest statement

The authors declare that the research was conducted in the absence of any commercial or financial relationships that could be construed as a potential conflict of interest.
